# Medication safety in family caregiving of older adults: home visits including interviews, observations, and medication reviews

**DOI:** 10.1186/s12877-026-07721-2

**Published:** 2026-05-29

**Authors:** Annika Kiiski, Marja Airaksinen, Eveliina Karasti, Shane Desselle, Marika Pohjanoksa-Mäntylä, Sirkka-Liisa Kivelä

**Affiliations:** 1 HUS Pharmacy, Helsinki University Hospital and University of Helsinki, Helsinki, 00029 Finland; 2 Division of Pharmacology and Pharmacotherapy, Faculty of Pharmacy, Clinical Pharmacy Group, University of Helsinki, PO Box 56, 00014 Helsinki, Finland; 3 Touro University College of Pharmacy, 11310 Club Dr, Vallejo, CA 94592 USA; 4 Institution of Family Medicine, University of Turku, Turku, 20014 Finland

**Keywords:** Medication error, Family caregiving, Home visits, Medication safety, Medication review, Older adults

## Abstract

**Background:**

Family caregivers, as non-professionals, have a responsible and challenging task, especially with older care recipients with multiple illnesses and complex treatments, including medications. This can lead to preventable risks and medication errors which can harm the care recipients.

**Objective:**

This study aimed to identify (1) medication safety risks, (2) medication errors, (3) contributing factors to the identified medication errors, and (4) the safeguards currently applied at home to avoid medication errors in family caregiving of older adults.

**Methods:**

The qualitative study involved 21 officially contracted family caregivers and their older (≥ 65 years) care recipients who used ≥ 1 prescription medicine recruited from the capital area of Finland. The data were collected during two consecutive home visits using: (1) thematic interviews, (2) observations, and (3) medication reviews during the period of October 2017 to September 2018. The data were qualitatively content analyzed by applying a deductive approach. Human error theory with systems approach and prospective risk management was used as a theoretical framework.

**Results:**

Medication safety risks and medication errors were found in all caregiving families. Most of the identified risks were related to administration, especially to characteristics of medications, medication taking techniques, behavior, and instructions. Medication errors occurred in storage, dispensing, administration, and follow-up. The most common identified medication errors were administration errors such as dose omission, wrong time, improper dose, and wrong duration. The most common contributing factors identified were related to administration errors. They included family caregiver-related factors (risky behavior), patient-related factors (e.g., behavioral factors such as non-compliance/non-adherence), organizational/service factors (e.g., medication use processes at home such as unsupervised medication taking), work/environmental factors (unlocked medication storage), and external factors (medication-related factors, lack of knowledge about medicines). As safeguards to prevent risks and medication errors, caregiving families commonly used pill dispensers, supervised medication taking, adjusted the medication taking schedule according to daily routines, used a cup to dispense medicines, and used a medication administration list.

**Conclusions:**

Medication safety risks and medication errors in family caregiving of older adults most related to the administration phase of the medication use process. Sufficient medicines information, information about medication management, up-to-date medication lists, locked medication storage, and enhanced pharmacists’ involvement in patient and caregiver education could be among actions needed for improving medication safety at home in family caregiving of older adults.

**Supplementary Information:**

The online version contains supplementary material available at 10.1186/s12877-026-07721-2.

## Keypoints

1. Medication risks and medication errors in family caregiving of older adults most commonly related to the administration phase of medication use process.

2. Sufficient medicines information, information about medication management, up-to-date medication list, locked medication storage, and enhanced pharmacists’ involvement in patient and caregiver education could be among actions needed for improving medication safety at home in family caregiving of older adults.

3. Locked storage is a small change at home but a powerful systemic defense against serious patient harm and therefore its importance should be highlighted to all caregivers in every possible training or education about medications or medication management.

**Supplementary Information:**

The online version contains supplementary material available at 10.1186/s12877-026-07721-2.

## Introduction

Family caregiving is an increasingly common way of taking care of patients needing long-term basic care which can be provided at home by non-professionals such as family members. Family caregiving is a demanding task requiring commitment and willingness to take on the holistic responsibility of the care recipient’s health and well-being. Caregiving for older adults is burdensome physically, mentally, and emotionally [1, 2]. As older adults often have multiple morbidities and complex polypharmacy to be managed at home, medication management is burdensome, demanding, binding, and contributes to family caregivers` overall stress [2, 3]. Caregivers as non-professionals may need to take such caregiving responsibility for which they are not prepared. This can lead to preventable risks and even medication errors harming the care recipients [4, 5]. However, systems and processes can be developed in any care setting to be more tolerant of human error to prevent error, reduce the chance of error, make it visible, or alleviate harm caused by errors [6].

Even though medication use is common in family caregiving, quite a few studies have focused on evaluating medication safety risks and errors in this context. Previous research has focused on the factors influencing medication management in family caregiving of older adults [7, 8]. These studies reported administration errors as side findings. The errors found were forgetting to take the medicine, taking the wrong medicine or taking the medicine at the wrong time, taking another person’s medication, or taking a wrong dose or multiple doses [7, 8]. The same studies identified some medication safety risks such as care recipients’ resistance in care situations, poor health and function of caregiver or care recipient (e.g., memory loss, difficulty swallowing or injecting medications, or dropping pills) and difficulty telling medications apart (lookalike medicines or a different generic medication). The safeguards reported in these studies were restricted access to medications, medication lists, pill boxes and other containers, checklists, calendars, and other reminder tools such as verbal reminders, notes, or alarm clocks, making modifications to pill boxes by highlighting, color coding, or writing on the box and using multiple pill boxes.

Previous research on medication errors at home has included all patient groups and proposes medication safety research should be targeted to home care, e.g., family caregiving [5, 7]. Previous studies on family caregiving have not systematically and in a theory-based manner focused on identifying medication errors, contributing factors, and safeguards in all phases of the medication use process at home involving family caregiving (storage, dispensing, administration, and follow-up). In addition, older adults form a high-risk patient segment for medication safety risks and errors in any care setting [9–13]. The need for evidence-based information about medication errors, medication safety risks, and risk management is evident, in order to improve the medication safety in older adults’ family caregiving at home. The aims of the study were to (1) identify medication safety risks and (2) medication errors in family caregiving of older adults, (3) identify contributing factors to the previously identified medication errors, and (4) identify the currently applied safeguards in family caregiving at home to avoid medication risks and errors.

## Methods

### Study context

This study was conducted in Finland in 2017 to 2018. At that time, approximately 48,000 family caregivers had made an official caregiver contract with the municipality [14, 15]. Municipalities paid contracted family caregiving families care benefits. The benefits were composed of the services provided to the care recipient (e.g., home care, daytime activity) and the care allowance (remuneration), caregivers’ days off (at least three days per month), and services supporting family care as defined in the contract (e.g., health checks and guidance and advice) [15]. According to the law, municipalities (nowadays well-being services counties) were obligated to give guidance and advice to ensure caregivers` capability to take care of the care recipient. Contracted family caregivers legally belonged to social services, but were truly positioned between formal and informal social- and healthcare service systems [2].

The family caregiving families varied according to the healthcare services they used, e.g., other families also had home care services and other families had the responsibility of medication management by themselves. In Finland, medication reviews every six months are listed as a quality indicator related to statutory services for older adults [14], but according to empirical knowledge the execution is lacking.

### Study design

**Fig. 1:**
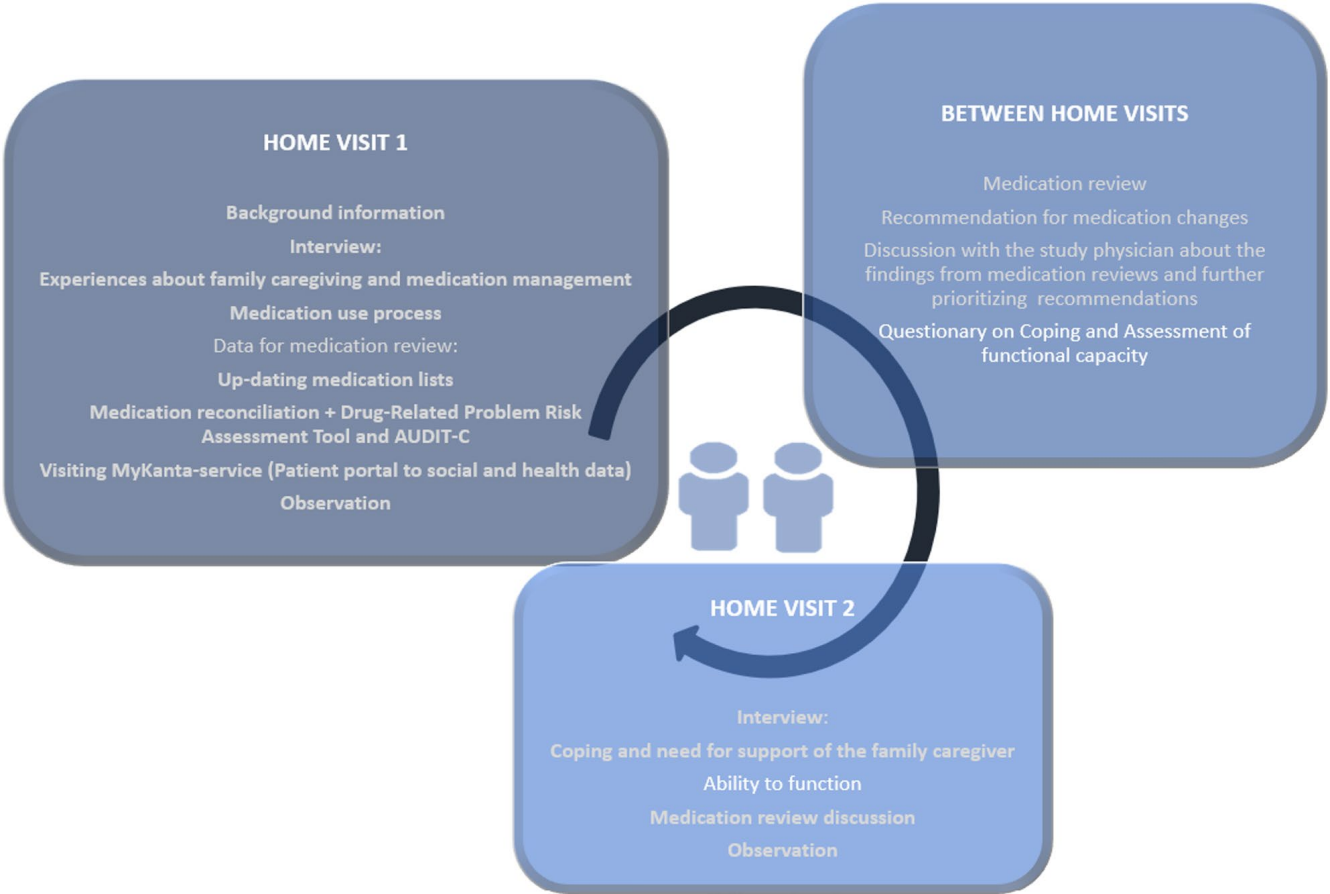
Study design with two consecutive home visits in family caregiving families (*n* = 21) using (1) interviews focusing on medication management practices at home, (2) observation, and (3) medication review. The data used in this study is marked in bold and grey in the figure

This study was conducted as method triangulation (Fig. 1) [16]. The data were collected during two consecutive home visits using (1) thematic interviews with narrative parts focusing on medication management practices at home, (2) observation during the home visits, and (3) medication review between the home visits based on data collected during visit 1, and the findings were discussed with the caregiver and care recipient during visit 2. Background information was collected using structured questionnaires before and during the first home visit. The present study is part of a larger research project: findings concerning caregivers’ challenges in engaging in health system to optimize medication management of older care recipients have been published elsewhere [17].

A systems approach to preventive risk management as presented in human error theory was used as a theoretical framework of the study [18]. Medication safety risks should be identified before they reach the patient and managed proactively by developing the healthcare system and its processes. This study sought such proactive risk management strategies in family caregiving at home by identifying both medication safety risks and medication errors that occurred.

### Participants

The volunteer caregiving families were recruited via the Association of Carers in the capital area of Finland in 2017 to 2018. Purposive selective sampling was used as a recruiting method. The following inclusion criterion were employed: (1) an older (*≥* 65 years) family care recipient, (2) at least one prescription medicine in use, and (3) the caregiver had an official caregiving contract with the municipality.

An announcement in a newspaper and recruitment letters were used to recruit caregiving families. The announcement in the newspaper of the Association of Carers in the capital area resulted in the recruitment of only three volunteer families. Therefore, recruitment letters were sent to two hundred randomly selected family caregiving families derived from the registered members of the Association of Carers in the capital area (yield: 18 families).

Registration for the study occurred through a telephone call to the main researcher (AK). During the call, the researcher verified that the inclusion criteria were met and scheduled the first home visit with the caregiving family. After the data obtained from the 21 home visits were analyzed, the need for additional data collection and recruitment of more caregiving families was evaluated in terms of data saturation. Saturation was considered to happen when collecting more data brought up no new theoretical views, nor revealed new theoretical categories [19]. As no new themes emerged during the analysis of the last two interviews, the material was considered saturated and no additional study participants were needed.

### Data collection and data used in this study

The data were collected during two consecutive home visits to volunteering caregivers (*n* = 21) and their care recipients (older adults over 65 years of age) in the capital area of Finland in 2017 to 2018 (Table 1). The study used method triangulation (Table 1) [16]. Background information was collected via structured questionnaires. Other data collection methods were:

1. Thematic interviews with caregivers (*n*=21) focusing on their narratives on managing medications of their care recipients at home (*n*=21) to understand the medication use process from their perspective.
2. Observation of medication management (e.g., storing of medications) and medication safety risks (e.g., crushing medicines, such as long-lasting depot formulations, that should not be crushed) in the caregiving families during the interviews and afterwards when observing the household.
3. Medication reviews (*n*=21) conducted of the care recipients and prescription reviews (*n*=21) (not as comprehensive as MR) conducted of the caregiver by the researcher.

**Table 1:** Collected data and data collection methods used in this study during the two consecutive home visits (*n* = 21)

DATA	DATA COLLECTION METHOD / DATA SOURCE	PHASE	DOCUMENTATION
MAIN DATA			
Interview transcript [3, 4, 20–24]	Discussion or interview	Home visits 1 and 2	Recording and transcript
Field notes	Observation	Home visits 1 and 2	Notes
Medication review interview	Discussion or interview	Home visits 1	Recording and transcript
Medication review discussion	Discussion or interview	Home visit 2	Recording and transcript and report
PATIENT INFORMATION USED FOR CONDUCTING A MEDICATION REVIEW			
Drug-related problem risk assessment tool [25, 26]	Discussion or interview	Home visit 1	Recording and transcript and form
Audit-C [27]	Discussion or interview	Home visit 1	Recording and transcript and form
Up-to-date medication lists	Discussion or interview	Home visit 1	Recording and transcript and form
Laboratory values	From the electronic patient record (Omakanta)[28]	Home visit 1	Form
Diagnoses	From the electronic patient record (Omakanta)[28]	Home visit 1	Form
BACKGROUND INFORMATION			
Cope-index [29]	Structured form	Between home visits	Form
Cope-index [29]	Discussion or interview	Home visit 2	Recording and transcript
Assessment of functional capacity (e.g. two questions about depression, self-assessed memory, ability to concentrate and ability to learn new things) [1, 30–34]	Discussion or interview	Home visit 2, between home visits	Recording and transcript, form
Other background information (e.g. the main reason for family caregiving, the need for healthcare services) [1]	Structured form	Before home visits	Forms

#### Home visits

The 21 family caregiving families were visited from October 2017 to September 2018. The principal researcher (AK) conducted all 42 home visits. The principal researcher (AK) had an MSc Pharm as well as several years of work experience at community pharmacies. In addition, pharmacists and experienced geriatricians (also an official family caregiver at the time of the study) belonged to the study group. One of the three pharmacy students assisted in making notes and observations in 40 of the 42 home visits. Each home visit lasted approximately two hours.

#### Thematic interviews

Interview guides were formed on the basis of systems thinking in medication safety [18] and using both semi-structured and narrative parts (Appendix 2 and 3). The same interviews were used in the earlier publication focusing on family caregiving families` challenges in engaging with the health system to optimize medication management [35]. The main themes in the first interview were: how family caregivers and care recipients managed with the everyday care and medication management, and medication use process [20, 23, 25–27, 35–37], and in the second interview: medication review discussion, caregiver’s coping and need for support, and both the caregiver’s and the care recipient`s functional ability [1, 20, 27, 29–35]. More specific questions were formed by using medication safety risks identified in the national guide for Safe Pharmacotherapy in social- and healthcare by the Ministry of Social Affairs and Health [21, 24] and the Family Caregiver Medication Administration Hassles scale [23].

Whether the care recipient and caregiver or only the caregiver participated in the interviews was defined by the disease and functional ability of the care recipient. A figure illustrating the medication use process was used as stimulus material for discussions [20, 36] (Appendix 1). The interviews were audio recorded and transcribed verbatim. The materials were confidentially handled, stored, and reported. The analysis and reporting of the study findings are anonymous, and hence conducted without the persons’ identification information to secure the protection of the study participants’ identity.

Two sets of pilot interviews were conducted to fine-tune study methods, pretest the interview guide, and to familiarize the main researcher (AK) with the study context. Minor clarifications were made and therefore pilots were included in the results.

#### Observations

Observations were conducted in the caregiving families during the interviews and afterwards when observing the household. Observations were documented in the field notes.

#### Medication reviews

Medication lists were updated for both caregiver and care recipient as background information for medication review and prescription review. If they did not have a medication list, one was compiled. The comprehensiveness and the type of the review (medication review or not such a comprehensive prescription review [38–41]) was determined according to the available patient information (whether there was access to the MyKanta service or not) [28]. Patient records (e.g., diagnosis and laboratory values) shared with patients are available in the MyKanta service (patient portal to social and health data). The first researcher (pharmacist, AK) reviewed the medications.

Medications were reviewed for possible burdens of adverse drug effects and adverse effects and interactions between medications. In addition, the purposes of medication, overlapping, dosages, duration of treatment, contraindications, pharmaceutical form, laboratory values, high risk medications (e.g., warfarin), patient adherence, success of self-care, medication-free treatment options, appropriateness of drug treatment reimbursements, prices, cheaper alternatives, storage of medications, expired medications, and the use of natural products were reviewed. Further, it was reviewed whether there is a medication for all diseases, medications prescribed without a diagnosis, or medications unsuitable for the older adults. The use of medications in renal failure and hepatic impairment, the prevalence of drug-induced diseases, and the correspondence of treatments to current care guidelines and monitoring (both at home monitoring and in social- and healthcare) were reviewed.

The research pharmacist (AK) sent the findings about medication to the research group’s geriatric medication expert (S-LK) and they held a meeting for each patient where they reviewed and prioritized the findings. If medication problems requiring physician attention were found, the physician responsible for the care was contacted by phone. One physician was contacted during the study by phone and letter and two nurses were phoned due to the acute problems in the care.

The summary of the medication review was supplemented with additional questions and recommendations for medication changes and were discussed with the families during the second home visit. With the permission of the care recipient, the summary and recommendations for medication changes were sent to the physician responsible for the care after the second interview. Summaries were sent in writing to the care recipient by mail after the last interview. The patient version was made easy to read without medical jargon. The medication review optimizes patients` medication, but in addition, in the review process, medication errors can also be identified (e.g., wrong dose, dose omission).

### Analyses

The data from interviews, observations, and medication reviews were analyzed by a qualitative content analysis applying a deductive approach partly using ATLAS.ti version 8.3.0 and Excel version 2016 [42]. A company specialized in transcription of audio records transcribed verbatim the caregiving families’ interviews for written text. Researchers (AK, EK) very familiar with the data conducted the analysis. They made the home visits and moderated the interviews during the visits.

The structured categorization matrix was made using Human error theory and multiple taxonomies. Human error theory and multiple taxonomies were used in deductive analyses to extract all the perspectives relating to the medication use management at home. Human error theory with a systems approach to preventive risk management were used as a theoretical framework for all the analyses (medication risks should be identified before they reach the patient and managed proactively by developing systems and processes). Human error theory and systems thinking guided the analysis and the presentation of results, e.g., with the use of fish bone diagrams for medication safety risks and contributing factors.

Data were categorized according to categorization matrix. The categories in the matrix were formed according to the multiple taxonomies. Categories for medication errors were constructed according to the National Coordinating Council for Medication Error Reporting and Prevention (NCC MERP) Taxonomy of Medication errors [43], the Conceptual Framework for the International Classification for Patient Safety from the World Health Organization (WHO) [44], and the medication error classification fish bone diagrams by the Ministry of Social Affairs and Health [21]. In addition, Ishikawa’s fishbone diagram method was used to illustrate medication error categorization as introduced in the medication safety context by the Ministry of Social Affairs and Health, Finland [21]. Contributing factors were categorized according to the Conceptual Framework for the International Classification for Patient Safety from the WHO [44]. Risk factors and safeguards were identified inductively but safeguards were categorized according to the Institute for Safe Medication Practices (ISMP) error prevention strategies and their power [6, 45].

Interview transcripts and field notes were analyzed according to a categorization matrix. Medication reviews were conducted as a part of the interviews, hence the medication errors and recommendations from medication reviews were included in the interview transcripts. All materials were analyzed using the same categorization matrix. Two researchers encoded data independently according to matrix. Cohen`s cappa was calculated for medication error types (storage, dispensing, administration, and follow-up errors), contributing factors, safeguards, and risks related different error types. Inter-rater reliability varied between 0.43 and 1, varying from moderate to almost perfect agreement.

Identified risks were not concretized as medication errors but could lead to one. Medication safety risks were identified prospectively and contributing factors retrospectively after errors happened. Hence, two separate terms are used here to distinguish the difference. Contributing factors related to medication errors were added to the fish bones in order to provide comprehensive understanding of medication safety risks in family caregiving of older adults. Risks, error, contributing factors, and safeguards have been presented according to the phases of medication use processes happening at home.

Based on the analysis, the categories were summarized in figures to describe the medication safety risks and medication errors, their contributing factors, and possible safeguards in the caregiving families:

1. Medication errors, and their contributing factors at home in family caregiving.
2. Possible safeguards at home in family caregiving and their power.
3. A fish bone diagram describing the risks when managing medications at home from the perspective of family caregivers and their care recipients.

The results have been first organized according to the study objectives at the overall level; medication errors, contributing factors, safeguards and medication safety risks. Medication safety risks have been presented last due to the fact that contributing factors related to medication errors were added to the fish bones of medication safety risks in order to provide comprehensive understanding of medication safety risks in family caregiving of older adults. Thereafter, medication errors, contributing factors, safeguards and medication safety risks have been presented below the phases of medication use process happening at home. Medication use process approach was applied to provide more comprehensive perspective e.g. what are the medication errors, contributing factors and medication safety risks related to the storage, and how to prevent errors from happening beforehand.

### Patient and public involvement

The Association of Carers in the capital area, Finland participated in the planning phase by commenting on the study design. The senior researcher (S-LK) was a contracted caregiver for her old spouse while the study was planned and conducted. Therefore, older care recipient and caregiver viewpoints were considered throughout the research process. Pilot interviews were used to build up methods and to familiarize the main researcher (AK) with the context.

## Results

The results have been organized according to the study objectives at the overall level. Thereafter, all phases of medication use process happening at home were assessed more thoroughly.

### Background information

Twenty-three caregiving families participated in the study (Table 2). Two of them were excluded after registration, yielding a total number of 21 participating caregiving families with an older care recipient. One family dropped out due to the care recipient’s death, and another was excluded since the care recipient’s age was under 65 years. The families have had the official family caregiving contract for an average of 5 years (range 0–20 years).

**Table 2:** Family caregiving families’ characteristics

**CAREGIVER**	
Age, years mean	77 (range 59–87)
All medications in use (prescription, over the counter, and medications used when needed)	4.5 (range 0–10)
Sex	n
• Female	12
• Male	9
Relation to care recipient	n
• Spouse	19
• Daughter	2
Occupation	n
• Retired	20
• Worker	1
Health status	n
• Excellent	2
• Good	4
• Moderate	15
Living arrangements	n
• In the same household	20
• Separately	1
CARE RECIPIENT	
Age, years mean	80 (range 65–96)
All medications in use (prescription, over the counter, and medications used when needed)	9.7 (range 3–16)
Sex	n
• Female	10
• Male	11
Reason for caregiving (one could choose multiple reasons)	n
• Memory impairment	13
• Deterioration of physical function	13
• Psychiatric illness	1
• Left-sided paralysis	1
Level of care the care recipient would need without caregiving (*n* = 20)	n
• Round-the-clock institutional care	9
• Assisted living (round-the-clock)	6
• Assisted living	1
• Home care service, visit also at night	2
• Home care during day times	2

In general, care recipients` and caregivers` medications were considered to meet the recommendations according to medication reviews. One patient had a severe serotonergic burden and three antipsychotic medications in use. Due to this, the physician was contacted during the study by phone and letter. Two nurses were phoned due to acute problems in the care; an automated dose dispensing (ADD) package was missing one medication and home care omitted to administer medications. Many of the administration errors were identified due to medication reviews.

### Medication errors, contributing factors, and used safeguards

**Fig. 2:**
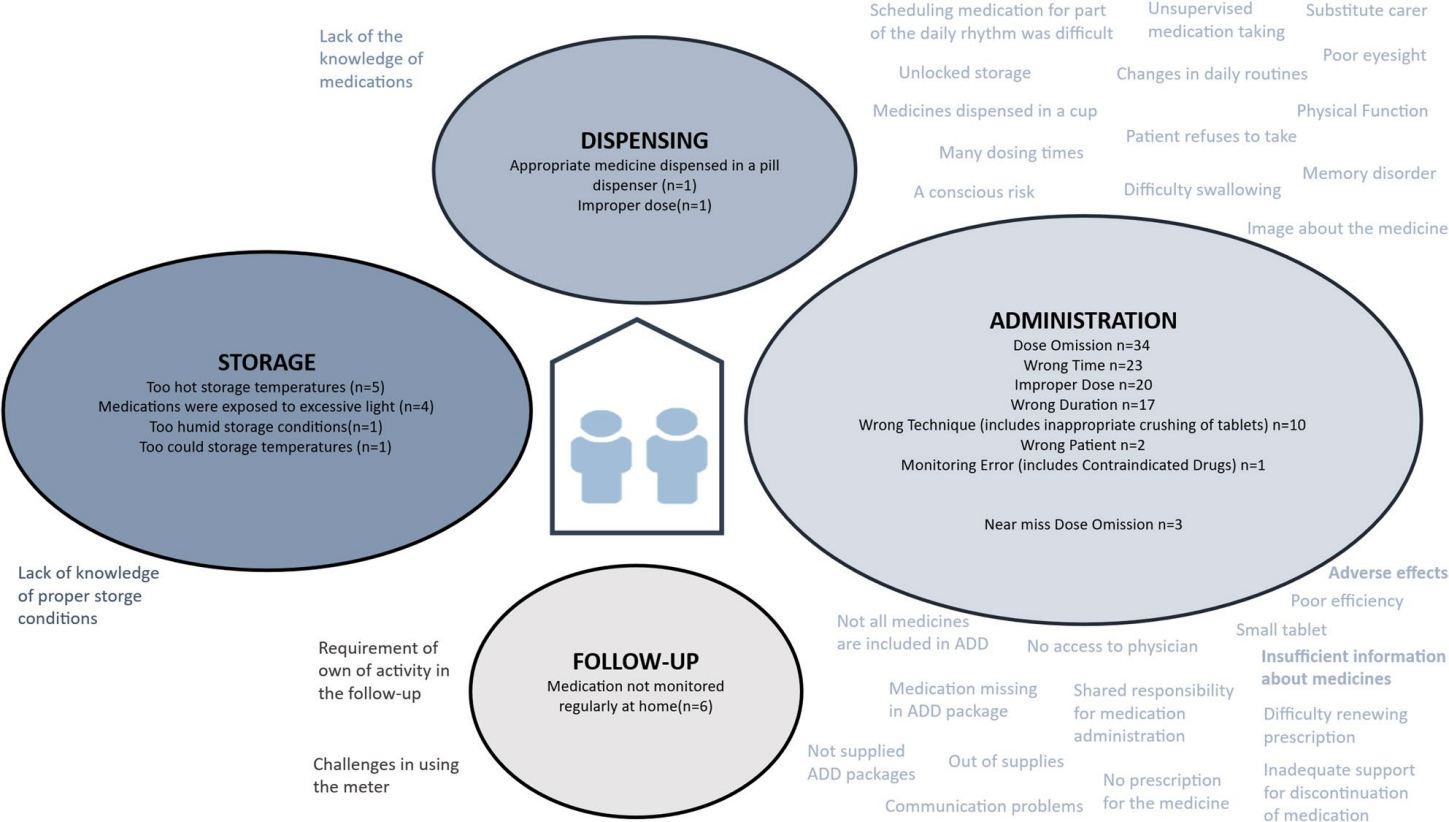
Medication errors (inside the circles) and contributing factors (outside the circles) at home in family caregiving older adults. The bolded contributing factors were the most common (ADD = automated dose dispensing)

A total of 126 medication errors were identified including all family caregiving families. Medication errors occurred in storage, dispensing, administration, and follow-up (Fig. 2). Most of the identified contributing factors were related to administration errors and the most common contribution factors were insufficient information about medications and adverse reactions. Both were external factors. Insufficient information about medications was related to the services category which family caregiving families received from healthcare and pharmacies. Adverse effects were related to products category here medications.

*“He didn’t say anything more about medication than that the screaming would stop.”* (Family 12).

Most of the safeguards used were related to dispensing and administration. Commonly, families used pill dispensers, supervised medication taking, adjustment of the medication taking as a part of the daily rhythm, a cup to dispense medications, and a list of medication administration as safeguards.

“*It’s just the same routine daily. In the morning*,* it’s related morning activities and in the evening*,* medicines are taken before going to bed*,* in the daytime X often rests during the day*,* medicines are taken after he gets up*.” (Family 2).

**Fig. 3:**
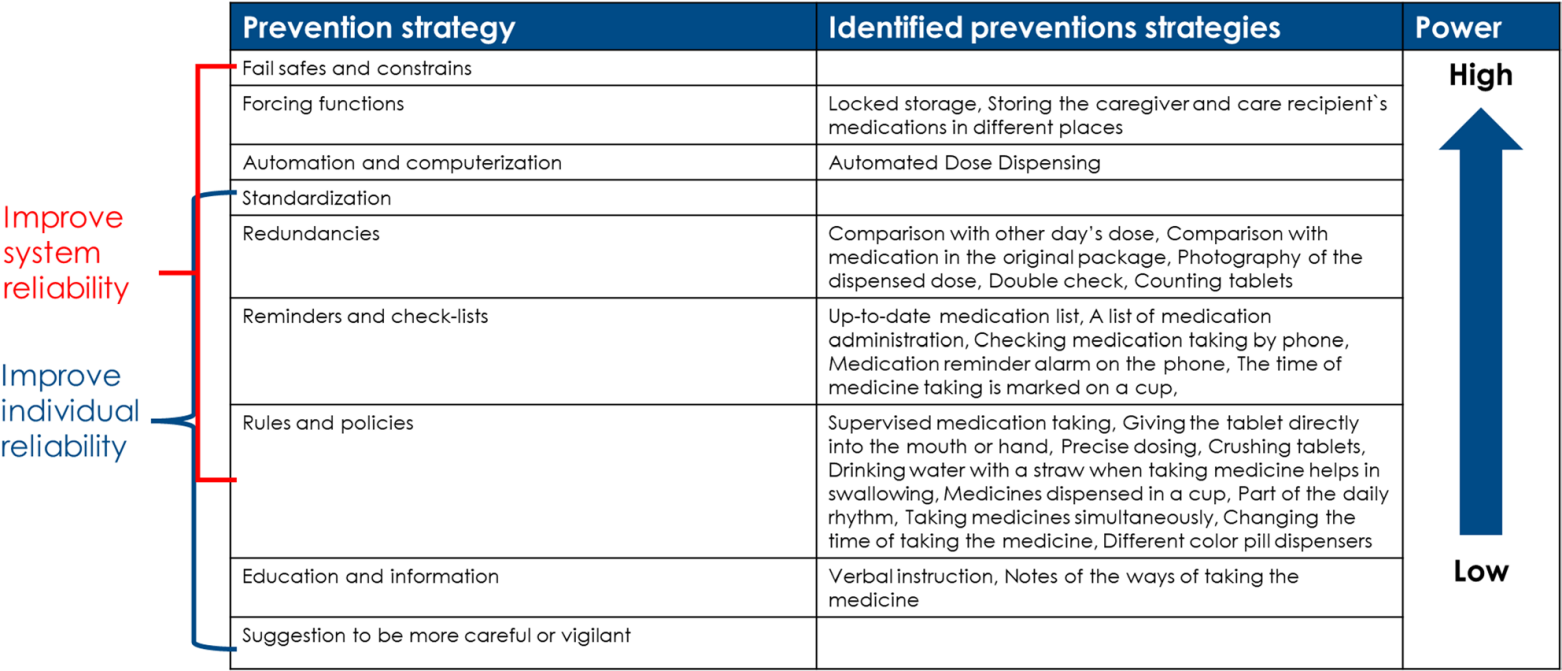
Safeguards used at home in family caregiving of older adults classified according to ISMP preventions strategies to review their power

Strategies related to storage were the most powerful strategies (Fig. 3). Most of the identified strategies were policies or practices used at home. Aids (tablet splitter, jar opener), pill dispensers, tools for opening the package, and medication-taking aids were not classifiable. Nonetheless, they can prevent medication omissions or wrong dose errors.

### Medication safety risks

**Fig. 4:**
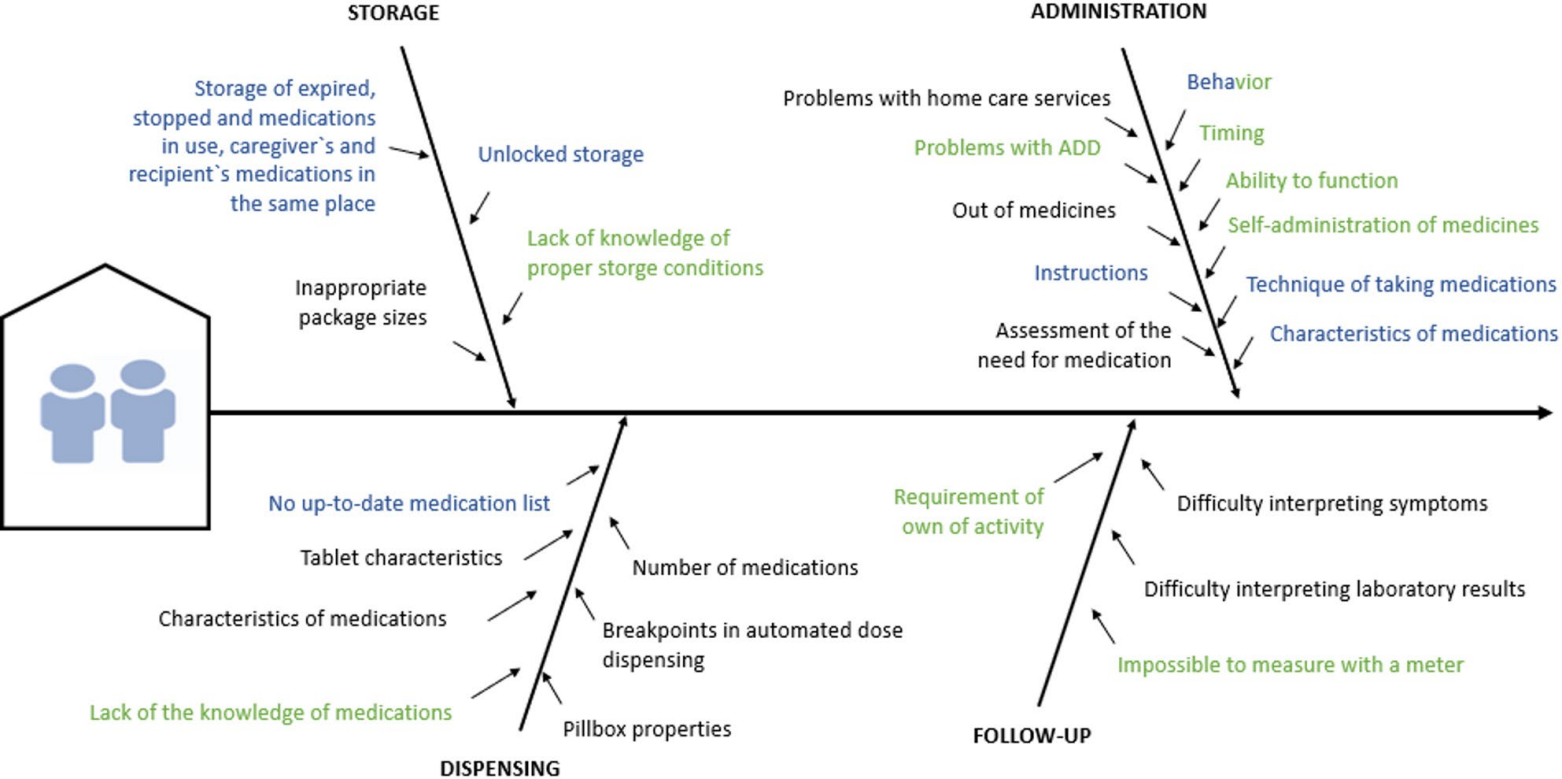
Medication safety risks at home in family caregiving of older adults. Contributing factors added as green, others identified by researchers not leading to medication error but could lead to one. Blue indicates those mentioned many times in the data (ADD = automated dose dispensing)

In addition to contributing factors, researchers identified medication safety risks in storage, dispensing, administration, and follow-up (Fig. 4). Most of the identified risks were related to administration and especially to characteristics of medications, technique of taking medications, behavior, and instructions.

“*Yes*,* sometimes he refuses to take the medicine*,* but very rarely*,* but sometimes it happens that he doesn’t want to take it*.” (Family 4).

### Storage

Due to the lack of knowledge about the correct storage conditions (contributing factor) for medications, the storage temperatures were too high (*n* = 5) or too cold (*n* = 1), medications were stored in too humid environments (*n* = 1), or the medicines were exposed to excessive light (*n* = 4). The lack of knowledge about the correct storage conditions was related to the category of healthcare and pharmacy services family caregiving families received, thus being an external factor. Caregiving families identified locked storage and storing the caregiver`s and care recipient`s medications in different places as safeguards in the storage conditions.

Identified medication safety risks in storage of the medications were unlocked storage, inappropriate package sizes (too big), and storing expired, stopped, and medications in use or caregivers` and recipients` medications in the same place. Several caregiving families justified the lack of locked storage as they felt no need for it, because small children did not visit them.

“*Well then*,* if we go back to that storage*,* where and how do you store the medicines? X: They’re in that closet*,* all mixed up. Y: I happily mess up old and new ones*.” (Family 1).

### Dispensing

Dispensing errors were identified in only a few (*n* = 2) caregiving families. One family identified the improper dose as the number of medications were wrong in the double check. In another family, medications which are sensitive to humidity and should be stored in the original package were dispensed in the pill dispenser. The contributing factor was lack of knowledge of medications and related to healthcare and pharmacy services family caregiving families received, thus being an external factor. Families used as safeguards for dispensing errors ADD, comparison with other days’ doses, comparison with medication in the original package, photography of the dispensed dose, aids (tablet splitter, jar opener), pill dispensers, up-to-date medication lists, different colored pill dispensers, double checking, and counting tablets.

Identified medication safety risks in dispensing were no up-to-date medication list, tablet characteristics (e.g., crumbling tablets, small size, lookalike tablets), characteristics of medications (e.g., using the medicine package is difficult, soundalike medications), number of medications, breakpoints in ADD while in hospital, and pillbox properties (e.g., compartments too small and markings wear off).

“*That’s the problem when they suddenly change medicines from another manufacturer*,* and they are no longer the same. And it’s such a hassle when there are a lot of little white pills that don’t really read anything like that*,* but we have them in packages over there*,* if nothing else*,* I’ll take the medicine package and see if it’s this one for sure.”* (Family 2).

### Administration

**Fig. 5:**
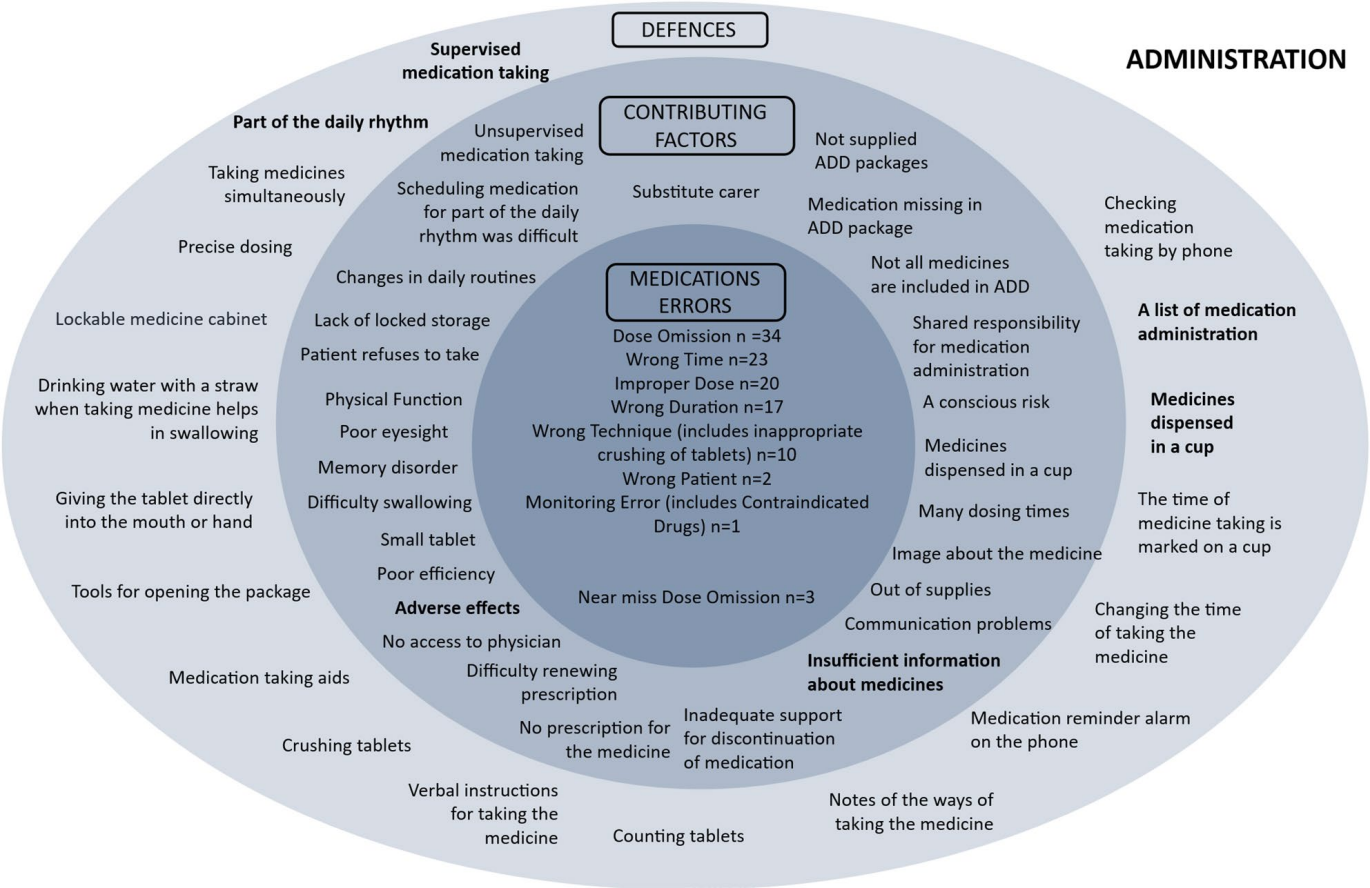
Medication errors, contributing factors, and defenses (safeguards) in family caregiving older adults when administrating medications at home. Bold indicates the most common ones

Medication errors occurred most commonly in administration of the medications (Fig. 5). Administration errors identified were:

1. Dose omission (*n*=34) (failure to administer (*n*=18) and failure to take (*n*=16))
2. Wrong patient (*n*=2)
3. Improper dose (*n*=20) (overdosage (*n*=8), under dosage (*n*=12))
4. Wrong time (*n*=23) (with food (*n*= 11), without food (*n*=1), wrong time (*n*=11))
5. Wrong duration *n*=17 (too long (*n*=2), stopped (*n*=15))
6. Monitoring error (includes contraindicated drugs) (*n*=1)
7. Wrong technique (*n*=10) (tablet halved (*n*=1), the patch came off (*n*=1), medicine crushed (*n*=2), medicine chewed (*n*=2), capsule opened (*n*=1), medicine diluted in water (*n*=2), mixed in food (*n*=1))

Near miss of dose omission (*n* = 3) was identified in three families (Fig. 5).

“*We had one problematic situation about a year and a half ago*,* when the sleep problems were at their worst*,* and the sleeping pills were on the top shelf of the kitchen cupboard. X had got a ladder and climbed up there*,* and found that package there and*,* I guess just took too many of them—was hospitalized for two weeks. After that*,* a locked medicine cabinet was bought to us”*. (Family 3)

Contributing factors identified in administration were related to family caregiver-related factors, patient-related factors, organizational/service factors, work/environmental factors, and external factors. The family caregiver-related factor was risky behavior. Patient-related factors were behavior (in more detail, non-compliance or non-adherence), cognitive factors (in more detail, perception or understanding) and pathophysiologic- or disease-related factors. Organizational or service factors were protocols, policies, or procedures (in more detail, medication use processes at home such as unsupervised medication taking). Work or environmental factors were physical environment or infrastructure such as unlocked medication storage. External factors were products and services (in more detail, medication-related factors or lack of knowledge about medicines). Identified safeguards were related to remembering to take medications, facilitating taking medications, and to prevent improper dose.

“*The neighbor’s dog was then offered tramadol solution. Her dog was then prescribed tramadol solution after the surgery. She asked whether you dared to try it when it was said that she was terribly sore. Then it was taken in varying amounts of 25–75 milliliters in drops*.” (Family 1).

**Fig. 6:**
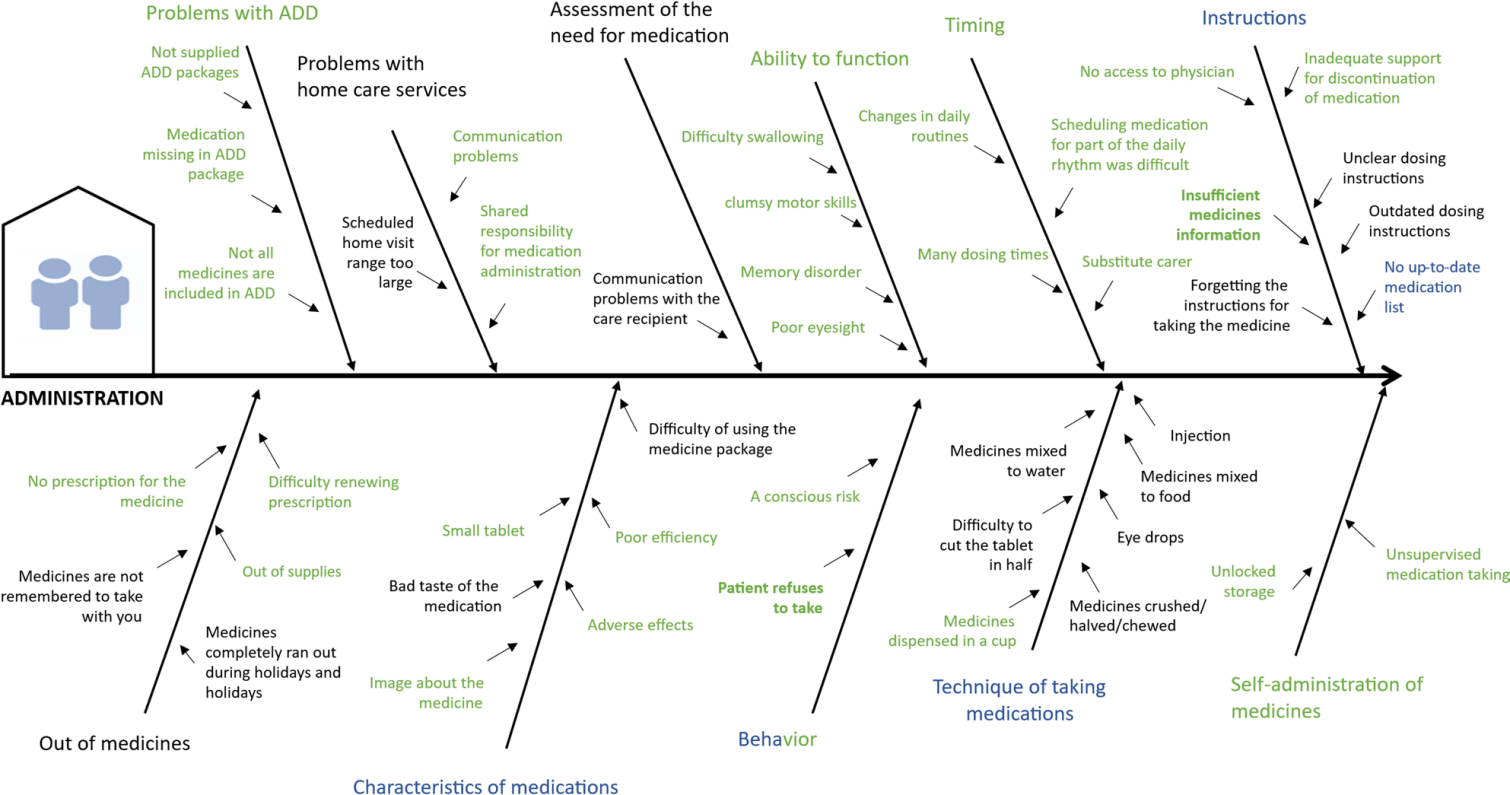
Medication safety risks in medication administration at home in family caregiving of older adults. Contributing factors added in green, others identified by researchers not leading to medication error but could lead to one. Blue indicates those mentioned many times in the data (ADD = automated dose dispensing)

Most common medication safety risks in medication administration were instructions, no up-to-date medication list, insufficient medicines information including characteristics of medications and technique of taking medications, and behavior, such as when the patient refuses to take the medication (Fig. 6).

“*No medication list*,* more in my head than what goes on regularly*.” (Family 11).

### Follow-up

Follow-up errors were medication not being monitored regularly at home (*n* = 6). Contributing factors related to medication errors were the requirement of own activity in the follow-up and challenges in using the meter. Requirement of own activity in the follow-up could be family caregiver-related factors (behavior: non-compliance) or external factor-related healthcare services. Challenges in using the meter is an external factor which was product related.

*“The follow-up phase is entirely up to the caregiver’s activity.”* (Family 14).

Identified medication safety risks in follow-up was difficulty of the interpretation of symptoms or laboratory values. No safeguards were identified in follow-up.

## Discussion

Comprehensive research methods with method triangulation and strong theory-orientation exposed more deeply the medication safety risks and errors of medication management at home. Medication errors occurred in storage, dispensing, administration, and follow-up, most commonly in administration. Due to this, the most common contributing factors and identified risk factors were related to administration errors. Families used multiple safeguards in order to prevent medication errors. These good practices should be utilized in all family caregiving families. Home visits and observation revealed medication safety risks that may concrete to medications errors. In order to manage these risks, attention should be paid to medicine information, up-to-date medications lists, locked medication storage, and information about medication management.

No wrong medicine or route errors were identified in our study. Otherwise, our findings are in line with previous findings. According to a previous systematic review including patients in all age groups, typical medication errors at home were forgetting to take the medicine, taking the wrong medicine or the wrong dose/strength, taking medicine at the wrong time, or route [5]. Wrong route errors were mainly present when administering medications to children. Previous studies in family caregiving of older adults reported medication errors as forgetting to take the medicine, taking the wrong medicine, or taking a medication at the wrong time, taking another person’s medication, or taking the wrong dose or multiple doses of a medication [7, 8]. In the healthcare context, most common administration errors were wrong time, dose, and omission errors [46]. Error types seem to be the same despite the context, although wrong duration error cannot be identified in the beginning of the treatment and are not so common in the hospital setting. Wrong duration errors can be detected by medication reviews which could also explain its occurrence.

In our study, identified contributing factors included family caregiver-related factors, patient-related factors, organizational/service factors, work/environmental factors, and external factors. A previous systematic review including patients in all age groups identified individual carer factors, environmental factors, medication factors, prescription communication factors, psychosocial factors, and care recipient factors as contributing factors [5]. All the others were also identified in our findings, besides the prescription communication factors. However, contributing factors were categorized differently due to the lack of a classification system in the systematic review. Prescription communication factors can be considered the same as lack of knowledge of medication. Hence, the underlying causes can be considered the same despite the age group.

As safeguards to prevent risks and errors, caregiving families commonly used pill dispensers, supervised medication taking, adjusted medication taking schedules according to daily routines, used a cup with which to dispense medicines, and employed a medication administration list in our study. Caregivers also used risk prevention strategies/safeguard that improve system reliability and can be considered more powerful strategies, such as locked storage. The more powerful strategies implemented in the system, the more likely error could be prevented. Similar safeguards were identified in previous studies. In family caregiving of older adults, the safeguards reported were restricted access to medications, medication list, pill box and other containers, checklists, calendars, other reminder tools such as verbal reminders, notes, or alarm clocks, making modifications to pill box by highlighting, color coding, or writing on it, and using multiple pill boxes [7, 8].

The health and function of caregivers were not prevalent in our data as a medication safety risk. This may be due to the recruiting technique and only active caregiving families attending the study. Otherwise, our findings are in line with the previous findings. Previous studies identified some medication safety risks such as resistance in care situations, health and function of caregiver, health and function of caregiver care recipients (e.g., memory loss, difficulty swallowing or injecting medications, or dropping pills) and difficulty telling medications apart (lookalike medicines or a different generic medication) [7, 8].

### Implications for practice

The present study indicates home visits conducted by professionals are vital in order to have comprehensive understanding about the problems in medication management at home in family caregiving of older adults and how families cope with them. In order to meet the needs of the family caregiving families, they should be better integrated into the healthcare [17]. With the help of healthcare professionals, medication management at home would be easier through learning and co-operation.

Lack of knowledge of medicines and their storage conditions were evident in different phases of medication use processes in our findings. According to the law, municipalities (nowadays well-being services counties) were obligated to give guidance and advice to ensure caregivers’ capability to take care of the care recipient. In addition, medicines information should be given in healthcare systems and pharmacies in order to ensure medication safety at home. The medicines information given by different healthcare professionals should be in line with and complement each other. According to a previous study, family caregivers experienced that their knowledge and skills in medication management improved with experience, but also described stress associated with information lag and gaps. Hence, family caregivers should receive better information, training, and support for this role [47]. Medicines information is the core know-how of pharmacists, therefore they should be more actively involved in the patient and caregiver education. For example, as a part of caregivers’ associations and local organizations organize guidance for caregivers, e.g., group lectures and municipalities/welfare areas counselling for caregivers and in hospitals as a part of the discharge process. The previous systematic review including patients in all age groups states that the effective means for preventing medication errors were carer training [5].

Up-to-date medication lists should be required before any medication can be administered. Lack of up-to-date medication lists and the knowledge about medications in use is evident in healthcare but also at home. All education for family caregivers should start with updating medication lists due to the fact that it is the basis of safe medication practices, and incorrect medications lists may lead to medication errors in diagnosis and implementation of medical treatment.

Unlocked medication storage is a risk for children but also self-destructive individuals or people with memory impairment. Locked storage is a small change at home but a powerful defense against serious patient harm. The importance of locked storage should be clarified for all caregivers in every training or education about medications or medication management.

### Strengths and weaknesses of the study

Due to the multimethod approach, strong theory base, and medication reviews, medication safety risks and errors were identified comprehensively. Through the unique qualitative research design, the study yields internationally and nationally new insights about medication safety at patients’ homes. Thus, the study adds valuable information to the academic medication safety risk management literature.

The rich qualitative data and observations ensured that the data supplemented other material, e.g., if families went through the medications used in the first phase, more medications were added to the list during the interview or observations at home. In light of the used methods, the sample size of 21 families can be considered sufficient [19, 48, 49] due to the fact that in the qualitative research the sample size needs to be small enough to undertake deep and comprehensive analysis. The data can be considered sufficient since it saturated by the eighteenth family. The triangulation method correlates data from multiple data collection methods and hence enhances the reliability of findings and ensures data saturation [16, 49]. The qualitative deductive content analysis employed, together with strong theory-oriented categorization and the prospective risk management framework ensured the data analysis was reproducible. Inter-rater reliability can be considered acceptable.

The recruited families may be active families due to the recruiting technique, which may reflect the results. The data collection method was time consuming due to two quite long consecutive home visits. Multiple error and contributing factor taxonomies were used as they were developed for the healthcare context, and none were comprehensive enough to capture the specific characteristics of family caregiving.

Taxonomy for the family caregiving context could be formed through, e.g., the Delphi method, in future studies. Future research should focus on different methods when studying medication errors, contributing factors, and used safeguards at home. E.g., observations studies could give more information about the medication errors, but the method is in dispute considering the venue is someone’s home. Factors causing medication burden have been reviewed as they may also be risks and contributing factors; however, medication burden was not assessed in the present study. Future studies should evaluate medication burden with a focus on medication management. To improve medication adherence, attention should be paid to family caregivers` knowledge of medications and health literacy in future research.

## Conclusions

Medication risks and errors in family caregiving of older adults most commonly related to the administration phase of medication use process. This study indicates that sufficient medicine information, information about medication management, up-to-date medication lists, locked medication storage, and enhanced pharmacist involvement in patient and caregiver education could be among actions needed for improving medication safety at home in family caregiving of older adults.

## Supplementary Information

Supplementary Material 1.

Supplementary Material 2.

Supplementary Material 3.

## Data Availability

The data that support the findings of this study are not openly available due to reasons of sensitivity and are available from the corresponding author (AK) upon reasonable request.

## Abbreviations

ADD: Automated Dose Dispensing
WHO: World Health Organization
