# Supplementary material for: Medication safety in family caregiving of older adults: home visits including interviews, observations, and medication reviews
Source: BMC Geriatr. 2026 May 29;26:1006. doi: 10.1186/s12877-026-07721-2 (PMC13435845; doi:10.1186/s12877-026-07721-2)

Appendix 1.
Medication risks and errors in family caregiving of older adults: Home visits including interviews, observations and medication reviews
Annika Kiiski, Marja Airaksinen, Eveliina Karasti, Shane Desselle, Marika Pohjanoksa-Mäntylä, Sirkka-Liisa Kivelä
Corresponding author: Annika Kiiski, Clinical Pharmacy Group, Division of Pharmacology and Pharmacotherapy, Faculty of Pharmacy, University of Helsinki and HUS Pharmacy, Finland, PO Box 56, 00014 University of Helsinki, Finland, ORCID: 0000-0003-3107-3854, Annika.kiiski@helsinki.fi

Appendix 1. Image of the medication use process [20,35]

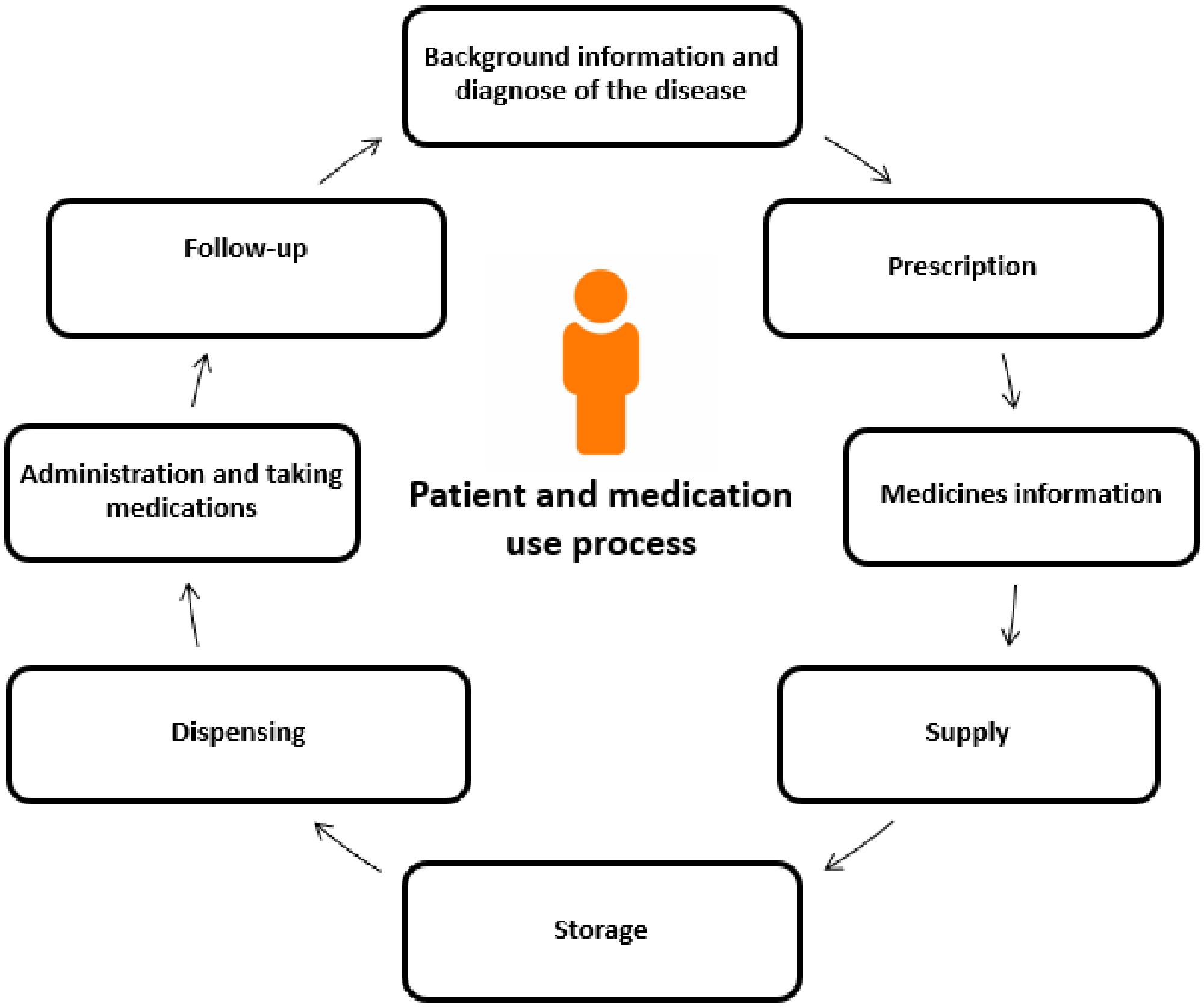

Supplement: Supplementary file 1 [file 12877_2026_7721_MOESM1_ESM.pdf]
