# Supplementary material for: Medication safety in family caregiving of older adults: home visits including interviews, observations, and medication reviews
Source: BMC Geriatr. 2026 May 29;26:1006. doi: 10.1186/s12877-026-07721-2 (PMC13435845; doi:10.1186/s12877-026-07721-2)
Supplement: Supplementary file 2 [file 12877_2026_7721_MOESM2_ESM.pdf]

Appendix 3.
Medication risks and errors in family caregiving of older adults: Home visits including interviews, observations and medication reviews
Annika Kiiski, Marja Airaksinen, Eveliina Karasti, Shane Desselle, Marika Pohjanoksa-Mäntylä, Sirkka-Liisa Kivelä
Corresponding author: Annika Kiiski, Clinical Pharmacy Group, Division of Pharmacology and Pharmacotherapy, Faculty of Pharmacy, University of Helsinki and HUS Pharmacy, Finland, PO Box 56, 00014 University of Helsinki, Finland, ORCID: 0000-0003-3107-3854, Annika.kiiski@helsinki.fi

CONDUCTING INTERVIEW 2

1. INTRODUCTION OF THE RECIPIENTS

- Introducing recipients (due to recording)

2. NEED FOR SUPPORT

- Do you generally feel well supported in your role as a family caregiver? (1)
    - Why yes / no?
- Where do you get support for medication management, what kind of support do you get?
    - Public healthcare and pharmacy
        - Describe cooperation and interaction with healthcare?
        - Describe cooperation and interaction with pharmacy?
        - Describe an experience where you were without support and had to make a difficult medication-related decision.
        - Where are the support services purchased from and who executed them (outsourcing)?
            - Is the level of services sufficient?
    - Organizations
    - Church
    - Social services
    - Friends or other relatives
        - Counseling
        - Volunteering
        - Peer support
- Where have you received information about diseases and medication? What have been the most important and reliable sources of information for you?(2)
    - Sources of information
        - Availability
        - Someone to ask(2)
        - Did you receive information in a timely manner?(2)
    - What to do if there are problems(2)
        - Adverse drug reactions
        - No effect
- Overall, do you feel well supported in your role as a caregiver?(1)
    - Why yes / no?
- Do you feel you cope well as a caregiver?(1)
    - Why yes / no?
    - What kind of coping methods do you have?
- Do you find caregiving too demanding?(1)
    - In what respect
    - What do you do when caregiving is too demanding?

Annika Kiiski, Marja Airaksinen, Eveliina Karasti, Shane Desselle, Marika Pohjanoksa-Mäntylä, Sirkka-Liisa Kivelä
Corresponding author: Annika Kiiski, Clinical Pharmacy Group, Division of Pharmacology and Pharmacotherapy, Faculty of Pharmacy, University of Helsinki and HUS Pharmacy, Finland, PO Box 56, 00014 University of Helsinki, Finland, ORCID: 0000-0003-3107-3854, Annika.kiiski@helsinki.fi

    - Did you lower the effectiveness or quality of caregiving when it was too demanding?
    - Did you change your perception of care recipient when the caregiving was too demanding?
    - Did your empathy for the person you were caring for increase or decrease at that time?
- Does caregiving have a negative effect on your physical health?(1)
    - How?
- Does caregiving cause you financial difficulties?(1)
    - How/where is this show?
- Do you feel trapped in your role as caregiver?(1)
    - Why?
- Do you find caregiving worthwhile?(1)
    - Why yes / no?
- Do you have a good relationship with the person you care for?(1)
    - How/where is this shown?
- Does caregiving have a negative effect on your emotional well-being?(1)
    - In what ways?
- Does caregiving cause difficulties in your relationship with your friends?(1)
    - What kind of difficulties?
- Does caregiving cause difficulties in your relationship with your family?(1)
    - What kind of difficulties?
- Do you feel well supported by friends or neighbors?(1)
    - What kind of support do they give?
- Do you feel supported by your family?(1)
    - What kind of support do they give?
- Do you feel well supported by health and social services (including those private services that are paid for by the municipality)?(1)
    - What kind of support? What should be more?
- Do you have to purchase health and social services that the municipality pays only partly in order to receive sufficient support?
    - What kind of support? What should be more?
- Do you have to purchase health and social services that the municipality does not pay in order to receive sufficient support?
    - What kind of support? What should be more?

3. ABILITY TO FUNCTION

- Are you satisfied with your life at the moment?
- What kind of physical condition do you have?
    - How do you maintain it?
    - Has family caregiving affected the maintenance of your physical condition?
- What kind of physical condition do your care recipient have?
    - How do you maintain it?
- How do you maintain your mental alertness?
    - Where do you find joy in life?

Annika Kiiski, Marja Airaksinen, Eveliina Karasti, Shane Desselle, Marika Pohjanoksa-Mäntylä, Sirkka-Liisa Kivelä
Corresponding author: Annika Kiiski, Clinical Pharmacy Group, Division of Pharmacology and Pharmacotherapy, Faculty of Pharmacy, University of Helsinki and HUS Pharmacy, Finland, PO Box 56, 00014 University of Helsinki, Finland, ORCID: 0000-0003-3107-3854, Annika.kiiski@helsinki.fi

- How do you maintain the mental health of your care recipient? What gives her/him joy in life?
- Does your care recipient have enough activities per day?
- Is the care recipient depressed, disinterested, or does he/she wish to die?(3)
- Would you be prepared for your care recipient to move to 24-hour care?
- What would you do (if you are not ready for it)?

4. MEDICATION REVIEW DISCUSSION

- Therapeutic interview
- Focuses on potential medication problems that arise from the medication review.
- Accessing MyKanta-service with consent by care recipient or caregiver, (patient portal to social and health data)

References:

1. McKee KJ, Philp I, Lamura G, Prouskas C, Oberg B, Krevers B, et al. The COPE index--a first stage assessment of negative impact, positive value and quality of support of caregiving in informal carers of older people. Aging Ment Health. 7. England2003. p. 39-52.
2. Travis SS, Bernard MA, McAuley WJ, Thornton M, Kole T. Development of the family caregiver medication administration hassles scale. Gerontologist. 2003;43(3):360-8.
3. Whooley MA, Avins AL, Miranda J, Browner WS. Case-finding instruments for depression. Two questions are as good as many. J Gen Intern Med. 1997;12(7):439-45.
