# Supplementary material for: Medication safety in family caregiving of older adults: home visits including interviews, observations, and medication reviews
Source: BMC Geriatr. 2026 May 29;26:1006. doi: 10.1186/s12877-026-07721-2 (PMC13435845; doi:10.1186/s12877-026-07721-2)
Supplement: Supplementary file 3 [file 12877_2026_7721_MOESM3_ESM.pdf]

Appendix 2.
Medication risks and errors in family caregiving of older adults: Home visits including interviews, observations and medication reviews
Annika Kiiski, Marja Airaksinen, Eveliina Karasti, Shane Desselle, Marika Pohjanoksa-Mäntylä, Sirkka-Liisa Kivelä
Corresponding author: Annika Kiiski, Clinical Pharmacy Group, Division of Pharmacology and Pharmacotherapy, Faculty of Pharmacy, University of Helsinki and HUS Pharmacy, Finland, PO Box 56, 00014 University of Helsinki, Finland, ORCID: 0000-0003-3107-3854, Annika.kiiski@helsinki.fi

BACKGROUND INFORMATION

1. INTRODUCTION

- Introducing researchers and study questions
  - The aim of this interview is to study your experiences on family caregiving and medication management
  - The purpose is to obtain caregiver-centered evidence to develop social and health services to meet your needs by sufficient support and education
  - Based on research data, the aim is to develop the implementation of drug treatment in family care and to provide sufficient and high-quality support and guidance to family caregivers.
  - The study is a part of emerita professor Sirkka-Liisa Kivelä's research project on the medication safety in the family caregiving.
  - The study is also a part of the medication safety and risk management wider study line of the clinical pharmacy group University of Helsinki
  - Your experiences are valuable
  - You have the opportunity to discuss your medications with the health care professional. Furthermore, your overall medication will be reviewed, and possible medication-related problems will be solved.

2. INTERVIEW

- Open discussion
- No right or wrong answers
- All aspects are valuable
- Looking for different experiences
- Depending on the diseases and functional ability of the care recipient, either the care recipient and caregiver or only the caregiver can participate in the interviews
- An interviewer asks questions, listens and takes care of timing
- Recording
- Notes
- Confidentiality during handling and reporting the results

3. SIGNING INFORMED CONSENT– two copies (one for recipients and one for interviewer)

CONDUCTING INTERVIEW

1. INTRODUCTION OF THE RECIPIENTS

- Introducing recipients (due to recording)

2. EXPERIENCES ABOUT FAMILY CAREGIVING

- What are your experiences of family caregiving?
- How have you ended up being a family caregiver?
  - Why
- What are your experiences of medication management at home?
  - How does the care recipient participate in the medication management?
  - Describe your roles
- Are you satisfied with medication?
  - Factors you are satisfied with
  - Factors you are NOT satisfied with

3. MEDICATIONS

- Up-dating medication list medicine by medicine thruogh conversation
  - How do you take your medications (medicines used, aims, indication, follow-up)?
  - Do you use any tools e.g. ADD automated dose dispenser, pill pox?
- Risk assessment tool for drug-related problems(1, 2) and AUDIT-C (3)

4. EXPERIENCES ON MEDICATIONS (Medication use process)

This figure shows different phases of the medication use process from prescribing to the follow-up. In the figure, the patient has been placed in the middle.(4, 5)

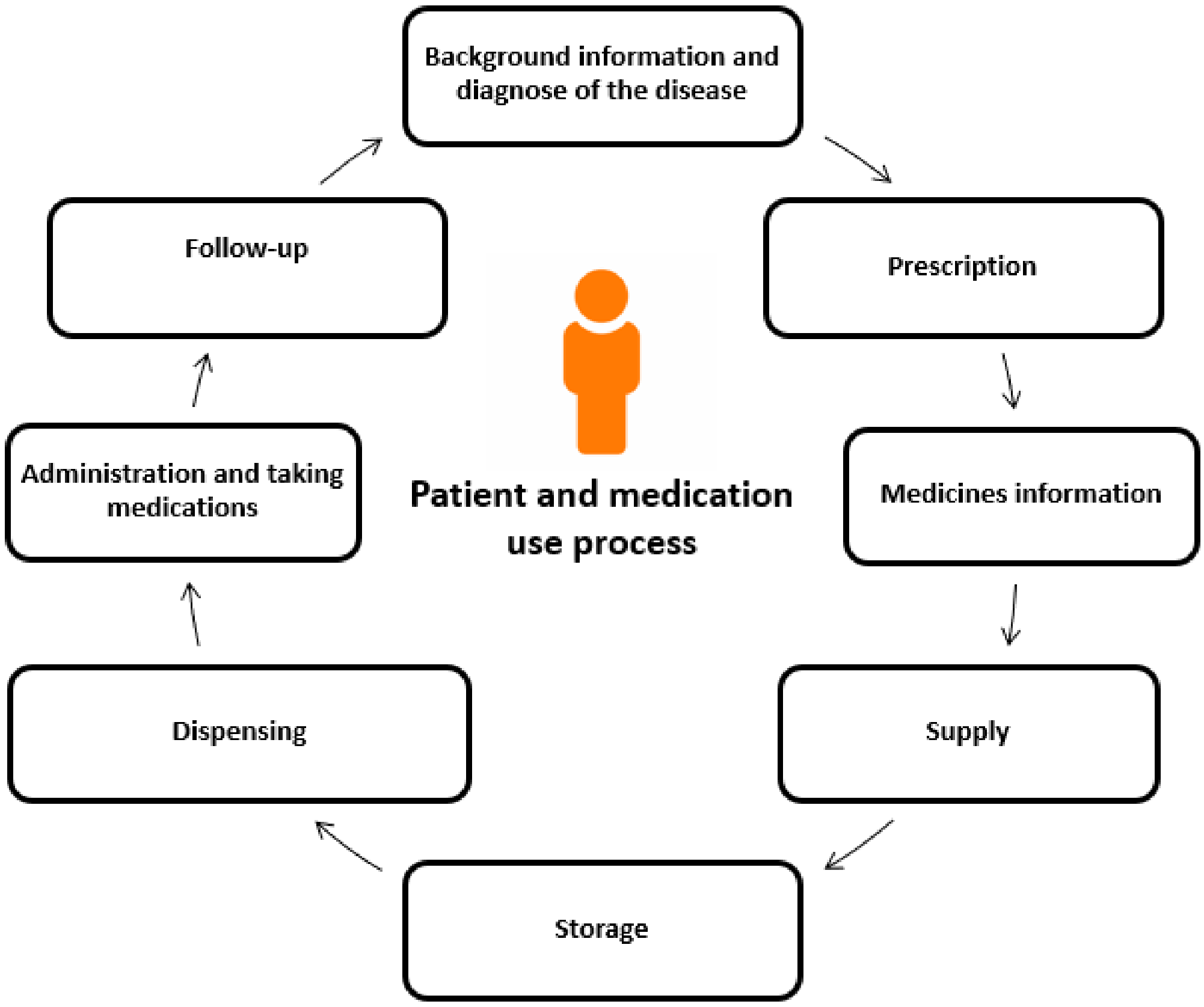

Next, we will go through the process figure together phase by phase. The following questions are asked in all phases:

- **How have the different stages of the medication use process been concretized in your life?**
- **Have you had any problems in the phases, what kind of, why and how they have been solved?** Process figure will be gone through together (Concrete examples, what has happened, solutions)
- **How should the different phases of the medication use process be like?** (Development ideas) How would you change different phases of the medication use process?

Next questions are asked under the titled phase:

- **Objectives of the medication**
  - Do you know the objectives of your medication? (6)
- **Identification of health problems and possible diagnoses**
  - How did the physician diagnose the health problem?
  - Has physician checked background information or made any clinical examination / tests before identification of health problem and making possible diagnoses?(7)
  - Was it easy for physicians to make diagnosis?
- **Prescribing**
  - Did the physician check
    - Possible allergies (7)
    - Other medicines in use (7)
    - Over-the-counter medicines in use(7)

        - Herbal products in use
        - Nutritional supplements in use
        - Homeopathic preparations in use
    - Did the physician provide information on (7)
        - The indication of the medication(6)
        - Therapeutic objectives of the medication (6)
        - When to expect the effects of the medication and what to expect(6)
        - Adverse drug effects and how to act if they occur
        - How to follow-up effects of the medication
    - What kind of advice did you expect to receive from the physician, but you didn't receive it?
    - Was it easy to discuss medication issues with the physician?(6)
    - Did you know what kind of questions to ask from the physician?(6)
- **Medicines information**
    - Where and from which sources have you received medicines information?
    - Have you been able to receive medicines information when in need of it?
    - What kind of advice did you expect to receive, but you didn't receive it?
    - About what topics would you like to have more medicines information?
    - Have you received sufficient information about the disease and the medication?
    - Has anyone told you about
        - Benefits and indication of your medication (6)
        - Duration of the medication
        - Adverse drug reactions(6)
            - how to act and whom to contact if they occur, or in case of other problems e.g. no effect from the medication. (6)
        - Objectives (6)
        - How to use medication at home(6)
        - Supporting self-care
        - When to expect effects
        - How to follow-up effects of the medication
        - What to do in case of missed doses
        - Understanding of the information received
- **Supply**
    - Where do you pick up your medications? (e.g. regular pharmacy)?
    - Have you run out of medications?(6)
        - During holidays(6)
    - Did the pharmacist provide you medicines information (see above)?(7)
    - Are the instruction labels adequate? (7)
    - Did the pharmacist check or counselled you about
        - Possible allergies (7)
        - Other prescription medicines in use(7)
        - Over the counter medicines in use (7)
        - Herbal products, nutritional supplements, homeopathic preparations in use
        - Prices of medicines and how to plan the medication purchases from an economic perspective
        - Reimbursement from Social Insurance
    - Are medicines too expensive to afford?
    - Do you have too many medications in use?
    - What kind of advice did you expect to receive from the pharmacy, but you didn't receive it?

    - What kind of support would you like to get from the pharmacy as a family caregiver? How could the pharmacy support you better than before?
- **Dispensing medication at home**
    - Who is responsible for dispensing your care recipient`s medications at home?
    - Do you use any tools e.g. pill dispenser?
- **Administration of medications (giving the medication)**
    - Is it difficult to schedule multiple medications per day?(7)
    - How do you schedule medicine taking as part of a daily routine? (6)
    - Is scheduling your own and your care recipients medications challenging?(6)
    - Do you give medications as scheduled? (6, 7)
    - How do you remember to give medications? (7) (6)
    - Is it easy to take medicines out of the package? If not, why?
    - How do you share roles in administration of medications?
    - Do you know when to increase or decrease dose or stop the medication? (6)
- **Administration of medications (taking the medication)**
    - Is it easy to take the medications (e.g. difficulty of swallowing, the use inhalations, or eye drops)?(7)
        - If there are problems with what medications?
    - How independently does the care recipient take medications? Describe how you share roles in medicine taking.
    - Do you halve, crush or chew medications? (6, 7)
    - Do you mix medications with food?
    - What liquid do you take medicines with?
    - Have you ever had any arguments with your care recipient about medicine taking? (6)
- **Storage**
    - Where and how do you store your medicines? (7)
    - Do the medications need special storage conditions at home?
- **Updating medication list**
    - What kind of medication list do you have in use (paper, Excel-table)?
    - Who updates the medication list, and how?
- **Follow-up**
    - How are the therapeutic objectives of medication monitored at home? Have you monitored them as a caregiver? (for example, blood pressure)?
    - How and where are the therapeutic objectives of the medications monitored? Do you have any follow-ups in healthcare (for example laboratory values)? What has been followed up? (7)
    - How are the follow-ups carried out? (7)
    - Have the medications been effective?
    - Have any adverse drug reactions occurred?
    - Have you followed adverse drug reactions together with healthcare professionals?
    - Who reconciles medications and reviews appropriateness of the medications?(7)
    - Has anyone conducted a comprehensive review of medications (e.g. physician or pharmacist)?(who, when and where)? (7)
- **Renewing prescriptions**
    - How the prescriptions are renewed? (6)
    - How is the further need for the medication confirmed?
    - How often does the care recipient see the physician?

- How do you feel about making medicines related decisions? (6)
- If a medication error, e.g. a missed dose, has occurred, has it been discussed with the physician?(6)

5. BACKGROUND VARIABLES

- Will be collected with structured form
- Accessing MyKanta-service with consent by care recipient or caregiver, (patient portal to social and health data)

1. Dimitrow MS, Leikola SN, Kivelä SL, Passi S, Lukkari P, Airaksinen MS. Feasibility of a practical nurse administered risk assessment tool for drug-related problems in home care. Scand J Public Health. 43. Sweden: © 2015 the Nordic Societies of Public Health.; 2015. p. 761-9.
2. Dimitrow MS, Mykkänen SI, Leikola SN, Kivelä SL, Lyles A, Airaksinen MS. Content validation of a tool for assessing risks for drug-related problems to be used by practical nurses caring for home-dwelling clients aged ≥65 years: a Delphi survey. Eur J Clin Pharmacol. 2014;70(8):991-1002.
3. Saunders JB, Aasland OG, Babor TF, de la Fuente JR, Grant M. Development of the Alcohol Use Disorders Identification Test (AUDIT): WHO Collaborative Project on Early Detection of Persons with Harmful Alcohol Consumption--II. Addiction. 1993;88(6):791-804.
4. Hakoinen S, Laitinen-Parkkonen P, Airaksinen M. [Lääkekaaoksen hallinta sote-muutoksessa – nykytila, haasteet ja ratkaisuehdotukset]. The Foundation for Municipal Development; 2017. Contract No.: Research series 106.
5. Mononen N, Pohjanoksa-Mäntylä M, Airaksinen MS, Hämeen-Anttila K. How far are we from a medication use process aiming at well-informed adherent patients with long-term medications in Finland? Qualitative study. BMJ Open. 2020;10(6):e036526.
6. Travis SS, Bernard MA, McAuley WJ, Thornton M, Kole T. Development of the family caregiver medication administration hassles scale. Gerontologist. 2003;43(3):360-8.
7. Ministry of Social affairs and Health. Safe Pharmacotherapy National guide for pharmacotherapy in social and health care Ministry of Social affairs and Health; 2009. Contract No.: Publications of the Ministry of Social Affairs and Health 2009:10.
